# Crystal structure of Pb_3_(IO_4_(OH)_2_)_2_

**DOI:** 10.1107/S1600536814009520

**Published:** 2014-06-23

**Authors:** Matthias Weil

**Affiliations:** a Institute for Chemical Technologies and Analytics, Division of Structural Chemistry, Vienna University of Technology, Getreidemarkt 9/164-SC, A-1060 Vienna, Austria

**Keywords:** crystal structure, lead, periodate, non-merohedral twinning

## Abstract

The structure of the title compound, trilead(II) bis­[di­hydroxido­tetra­oxido­iodate(VII)], was determined from a crystal twinned by non-merohedry with two twin domains present [twin fraction 0.73 (1):0.27 (1)]. It contains three Pb^2+^ cations and two IO_4_(OH)_2_^3−^ anions in the asymmetric unit. Each of the Pb^2+^ cations is surrounded by eight O atoms (cut-off value = 3.1 Å) in the form of a distorted polyhedron. The octa­hedral IO_4_(OH)_2_^3−^ anions are arranged in rows extending parallel to [021], forming a distorted hexa­gonal rod packing. The cations and anions are linked into a framework structure. Although H-atom positions could not be located, O⋯O distances suggest medium-strength hydrogen-bonding inter­actions between the IO_4_(OH)_2_ octa­hedra, further consolidating the crystal packing.

## Abstract 2

The basic building units of the hydrous periodate Pb_3_(IO_4_(OH)_2_)_2_ are three Pb^2+^ cations and two IO_4_(OH)_2_^3−^ anions. The octa­hedral anions are arranged in a distorted hexa­gonal rod packing, with the cations (each with a coordination number of eight) located in between.

## Chemical context   

Lead and mercury can both exist in different oxidation states and each of the two elements exhibits a peculiar crystal chemistry. In the case of Pb^2+^-containing compounds, the crystal chemistry is mainly dominated by the stereoactive 6*s* ^2^ lone-pair of lead (Holloway & Melnik, 1997 ▶), whereas Hg^2+^-containing compounds show a strong preference for a linear coordination of mercury (Breitinger, 2004 ▶). In this respect, it appears surprising that for some Pb^2+^- and Hg^2+^-containing compounds an isotypic relationship exists, *e.g.* for PbAs_2_O_6_ (Losilla *et al.*, 1995 ▶) and HgAs_2_O_6_ (Mormann & Jeitschko, 2000*b* ▶; Weil, 2000 ▶), or for the mineral descloizite PbZn(VO_4_)OH (Hawthorne & Faggiani, 1979 ▶) and the synthetic phase HgZn(AsO_4_)OH (Weil, 2004 ▶). With this in mind, it seemed inter­esting to study the relation between phases in the systems Hg^II^–I^VII^–O–H and Pb^II^–I^VII^–O–H. Whereas in the system Hg^II^–I^VII^–O–H two compounds have been structurally characterized, *viz*. Hg_3_(IO_4_(OH)_2_)_2_ (Mormann & Jeitschko, 2000*a* ▶) and Hg(IO_3_(OH)_3_) (Mormann & Jeitschko, 2001 ▶), a phase in the system Pb^II^–I^VII^–O–H has not yet been structurally determined, although several lead(II) periodate phases have been reported to exist. Willard & Thompson (1934 ▶) claimed to have obtained only one phase with composition Pb_3_H_4_(IO_6_)_2_ in the system Pb^II^–I^VII^–O–H. However, Drátovský & Matějčková (1965**a* ▶,b* ▶) reported the existence of three phases with composition Pb_3_(IO_5_)_2_·H_2_O, Pb_2_I_2_O_9_·3H_2_O and Pb_4_I_2_O_11_·5H_2_O in this system. To shed some light on the conflicting composition of the Pb:I 3:2 phase [Pb_3_H_4_(IO_6_)_2_ *versus* Pb_3_(IO_5_)_2_·H_2_O with a lower water content], the synthetic procedure described by Willard & Thompson (1934 ▶) was repeated for crystal growth of this lead periodate. The current structure determination of the obtained crystals showed the composition Pb_3_H_4_(IO_6_)_2_ as reported by Willard & Thompson (1934 ▶) to be correct. In a more reasonable crystal–chemical sense, the formula of these crystals should be rewritten as Pb_3_(IO_4_(OH)_2_)_2_.

## Structural commentary   

**Figure 1:**
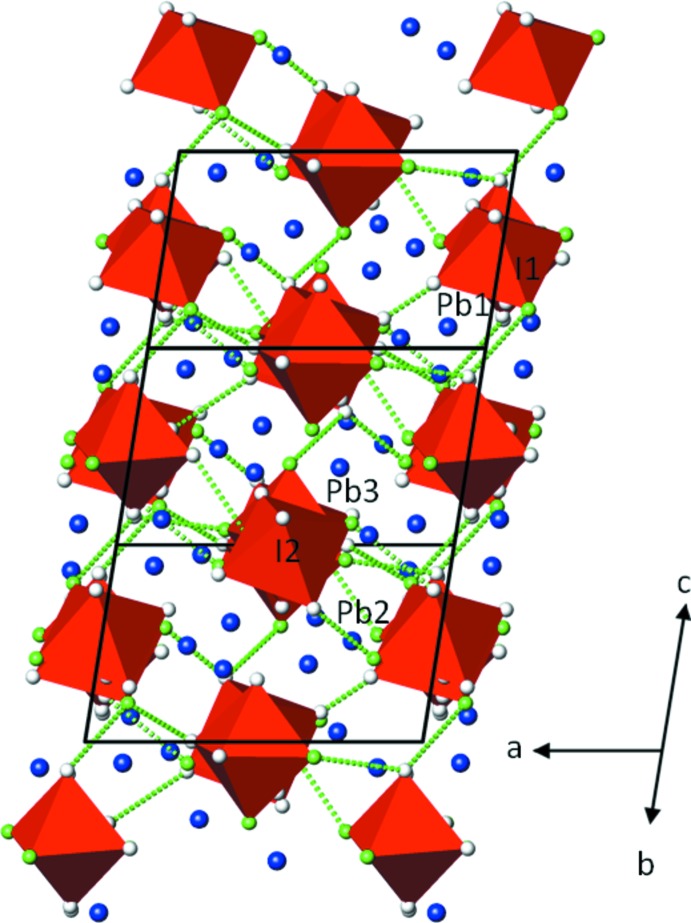
The crystal structure of Pb_3_(IO_4_(OH)_2_)_2_ in a projection along [021]. Displacement ellipsoids are drawn at the 90% probability level. O atoms bearing the OH function are given in green, the other O atoms are white. Pb—O bonds are omitted for clarity; hydrogen-bonding inter­actions are shown as green dashed lines.

Three Pb^2+^ cations and two IO_4_(OH)_2_^3−^ octa­hedra are present in the asymmetric unit. The anions form a slightly distorted hexa­gonal rod packing with the rods extending parallel to [021]. Cations and anions are linked through common oxygen atoms into a framework structure (Fig. 1 ▶).

**Figure 2:**
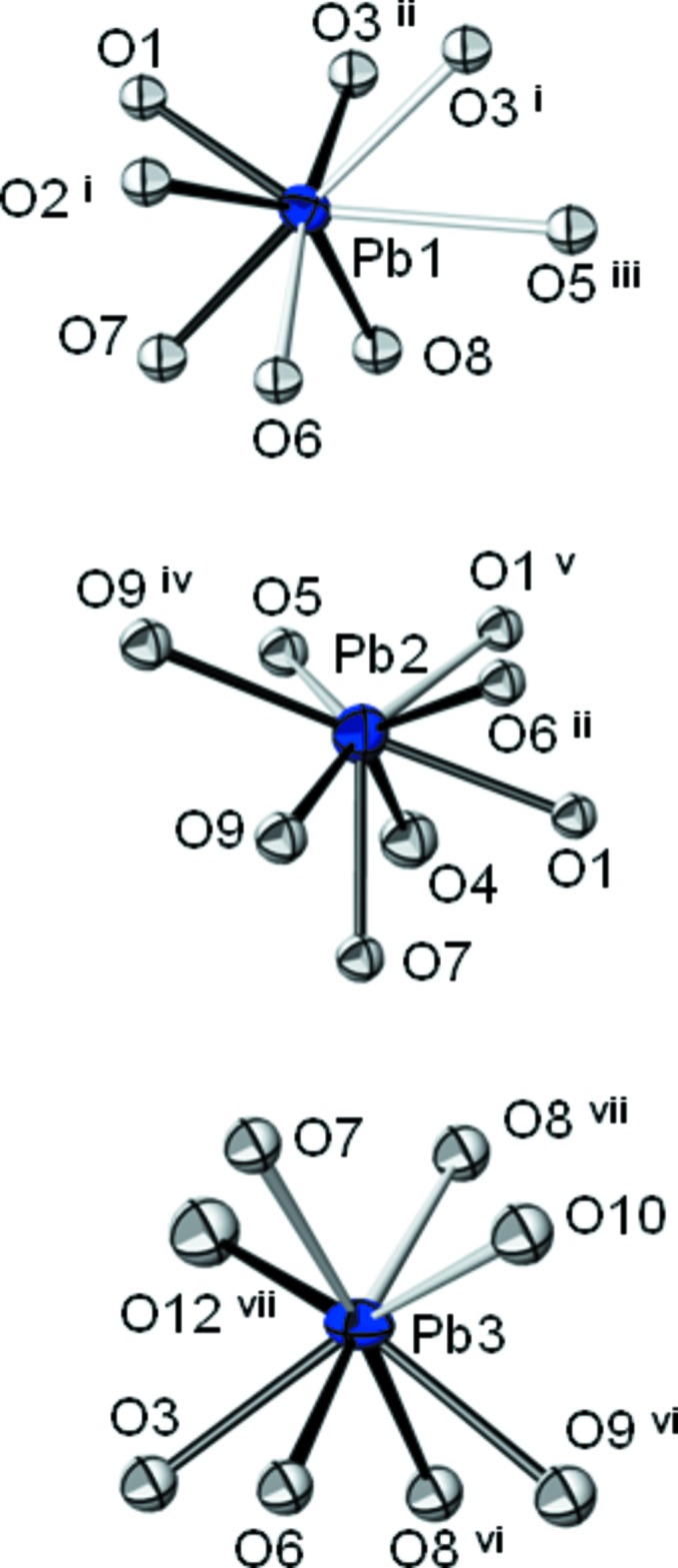
Coordination polyhedra of the three Pb^2+^ cations in the structure of Pb_3_(IO_4_(OH)_2_)_2_. Bonds shorter than 2.7 Å are given by solid black lines, longer bonds between 2.7 and 3.1 Å as open black lines. Displacement ellipsoids are drawn at the 90% probability level. [Symmetry codes: (i) −*x*, *y* − 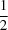, −*z* + 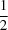; (ii) *x*, −*y* + 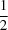, *z* − 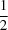; (iii) *x*, *y* − 1, *z*; (iv) −*x* + 1, −*y* + 1, −*z*; (v) −*x*, −*y* + 1, −*z*; (vi) *x*, −*y* + 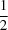, *z* + 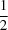; (vii) −*x* + 1, *y* + 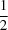, −*z* + 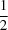; (viii) −*x*, *y* + 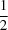, −*z* + 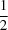.]

Each of the Pb^2+^ cations exhibits a coordination number of eight if Pb—O inter­actions less than 3.1 Å are considered to be relevant. The resulting [PbO_8_] polyhedra are considerably distorted [Pb—O distances range from 2.433 (7) to 3.099 (8) Å]. The stereochemical activity of the electron lone pairs in each of the polyhedra appears not to be very pronounced (Fig. 2 ▶).

Compounds and structures containing the periodate anion have been reviewed some time ago by Levason (1997 ▶). The compiled I—O bond lengths are in good agreement with the two IO_6_ octa­hedra of the title compound, having a mean I—O distance of 1.884 Å. Very similar mean values are found for comparable periodate compounds with large divalent cations, for example in BaI_2_O_6_(OH)_4_·2H_2_O (one IO_6_ octa­hedron, 1.895 Å; Häuseler, 2008 ▶), in Ba(IO_3_(OH)_3_) (one IO_6_ octa­hedron, 1.879 Å; Sasaki *et al.*, 1995 ▶), in Hg_3_(IO_4_(OH)_2_)_2_ (two IO_6_ octa­hedra, 1.888 Å; Mormann & Jeitschko, 2000*a* ▶) or in Sr(IO_2_(OH)_4_)_2_·3H_2_O (two IO_6_ octa­hedra, 1.888 Å; Alexandrova & Häuseler, 2004 ▶).

Results of bond-valence calculations (Brown, 2002 ▶), using the parameters of Brese & O’Keeffe (1991 ▶) for I—O bonds and of Krivovichev & Brown (2001 ▶) for Pb—O bonds, are reasonably close to the expected values (in valence units): Pb1 1.89, Pb2 1.73, Pb3 1.89, I1 6.78, I2 6.90, O1 1.95, O2 1.49, O3 1.90, O4 1.15, O5 1.15, O6 1.92, O7 1.98, O8 1.95, O 9 1.97, O10 1.09, O11 1.34, O12 1.12. The O atoms involved in hydrogen bonding are readily identifiable. The donor O atoms O4, O5, O10 and O12 exhibit the longest I—O bonds and the lowest bond-valence sums. Atom O11 has also a low bond-valence sum, explainable by its role as a twofold acceptor atom of medium-strength hydrogen-bonding inter­actions (Table 2 ▶) that additionally stabilize the packing of the structure (Fig. 1 ▶).

**Figure 3:**
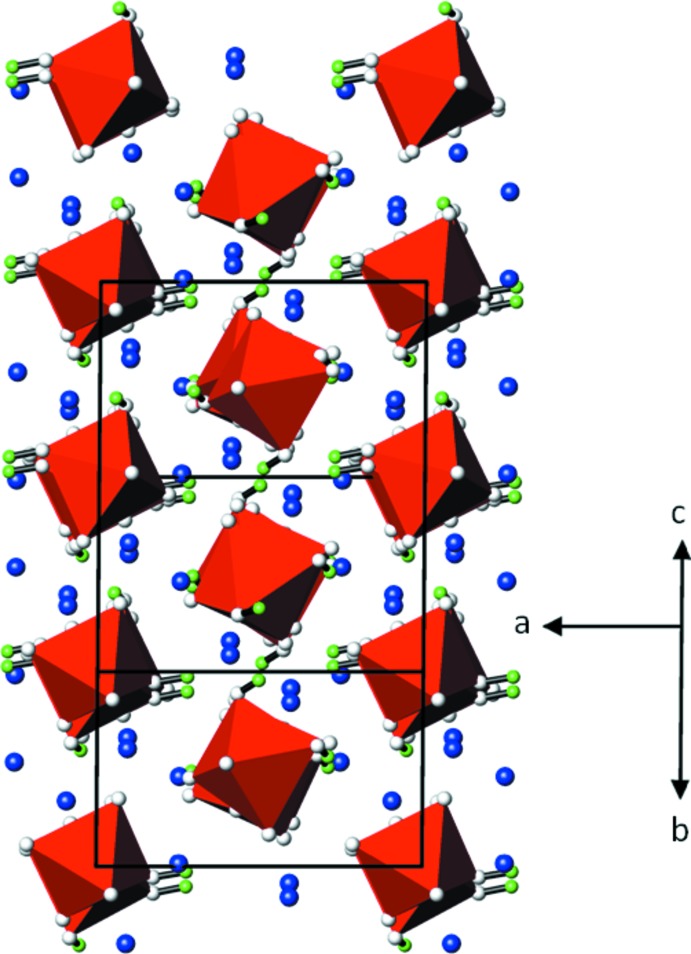
The crystal structure of Hg_3_(IO_4_(OH)_2_)_2_ (Mormann & Jeitschko, 2000*a* ▶) in a projection along [011]. Colour code as in Fig. 1 ▶. Hg—O and O—H⋯O inter­actions are omitted for clarity.

Comparison of the structures of Pb_3_(IO_4_(OH)_2_)_2_ and of Hg_3_(IO_4_(OH)_2_)_2_ [*P*2_1_/*c*; *Z* = 4, *a* = 8.5429 (7), *b* = 12.2051 (8) Å, *c* = 9.3549 (8) Å, *β* = 90.884 (7)°] reveals some close similarities. A ‘true’ isotypic relationship (Lima-de-Faria *et al.*, 1990 ▶) is difficult to derive for the two structures. However, they are isopointal and show the same type of arrangement in terms of the crystal packing. In the mercury compound, the IO_4_(OH)_2_^3−^ octa­hedra are likewise hexa­gonally packed in rods (Fig. 3 ▶). The cations are situated in between this arrangement which is further consolidated by O—H⋯O hydrogen bonding.

## Synthesis and crystallization   

The preparation conditions described by Willard & Thompson (1934 ▶) were modified slightly. Instead of using NaIO_4_ as the periodate source, periodic acid was employed.

1.25 g Pb(NO_3_)_2_ was dissolved in 25 ml water, acidified with a few drops of concentrated nitric acid and heated until boiling. Then the periodic acid solution (0.85 g in 25 ml water) was slowly added to the lead solution. The addition of the first portion of the periodic acid solution (*ca.* 3–4 ml) resulted in an off-white precipitate near the drop point that redissolved under stirring. After further addition, the precipitate remained and changed the colour in the still boiling solution from off-white to yellow–orange within half an hour. After filtration of the precipitate, a few colourless crystals of the title compound formed in the mother liquor on cooling. X-ray powder diffraction data of the polycrystalline precipitate are in very good agreement with simulated data based on the refinement of Pb_3_(IO_4_(OH)_2_)_2_.

## Refinement   

All investigated crystals were twinned by non-merohedry. Intensity data of the measured crystal could be indexed to belong to two domains, with a refined twin domain ratio of 0.73 (1):0.27 (1). Reflections originating from the minor component as well as overlapping reflections of the two domains (less than 10% of all measured reflections) were separated and excluded. The H atoms of the IO_4_(OH)_2_ octa­hedra could not be located from difference maps and were therefore not considered in the final model. The O atoms were refined with isotropic displacement parameters. The remaining maximum and minimum electron densities are found 0.73 and 0.68 Å, respectively, from atom Pb2. Structure data were finally standardized with *STRUCTURE-TIDY* (Gelato & Parthé, 1987 ▶). It should be noted that the intensity statistics point to a pronounced *C*-centred basis cell (space group *C*2/*c* with lattice parameters of *a* ≃ 14.16, *b* ≃ 9.21, *c* ≃ 8.97 Å, *β* ≃ 117.4°) with weak superstructure reflections violating the *C*-centering.

## Supplementary Material

Crystal structure: contains datablock(s) I, global. DOI: 10.1107/S1600536814009520/hb0004sup1.cif

Structure factors: contains datablock(s) I. DOI: 10.1107/S1600536814009520/hb0004Isup2.hkl

CCDC reference: 1004265

Additional supporting information:  crystallographic information; 3D view; checkCIF report

## Figures and Tables

**Table 1:** Selected bond lengths (Å)

I1—O6	1.845 (8)	I2—O11	1.820 (8)
I1—O3	1.860 (7)	I2—O9	1.850 (8)
I1—O2^i^	1.861 (7)	I2—O8	1.855 (7)
I1—O1^ii^	1.877 (7)	I2—O7	1.874 (8)
I1—O5^i^	1.920 (8)	I2—O12	1.932 (9)
I1—O4^i^	1.956 (8)	I2—O10	1.954 (8)

Symmetry codes: (i) 
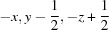
; (ii) 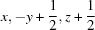.

**Table 2:** Hydrogen-bond geometry (Å)

*D*—H⋯*A*	*D*⋯*A*	*D*—H⋯*A*	*D*⋯*A*
O4⋯O7	2.849 (11)	O10⋯O11^iv^	2.675 (11)
O4⋯O2^i^	2.849 (11)	O12⋯O2^iv^	2.852 (11)
O5⋯O11^iii^	2.634 (11)		

Symmetry codes: (i) 
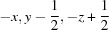
; (iii) 
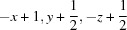
; (iv) 
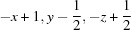
.

**Table 3:** Experimental details

Crystal data	
Chemical formula	Pb_3_[IO_4_(OH)_2_]_2_
*M*_r_	1071.40
Crystal system, space group	Monoclinic, *P*2_1_/*c*
Temperature (K)	296
*a*, *b*, *c* (Å)	8.9653 (9), 9.2113 (9), 12.8052 (13)
β (°)	101.042 (2)
*V* (Å^3^)	1037.90 (18)
*Z*	4
Radiation type	Mo *K*α
μ (mm^−1^)	54.55
Crystal size (mm)	0.06 × 0.06 × 0.05

Data collection	
Diffractometer	Siemens *SMART* CCD
Absorption correction	Multi-scan (*TWINABS*; Bruker, 2008 ▶)
*T*_min_, *T*_max_	0.253, 0.746
No. of measured, independent and observed [*I* > 2σ(*I*)] reflections	3196, 3196, 2587
(sin θ/λ)_max_ (Å^−1^)	0.716

Refinement	
*R*[*F* ^2^ > 2σ(*F* ^2^)], *wR*(*F* ^2^), *S*	0.041, 0.087, 1.07
No. of reflections	3196
No. of parameters	94
H-atom treatment	H-atom parameters not refined
	
Δρ_max_, Δρ_min_ (e Å^−3^)	2.88, −1.95

Computer programs: *SMART* (Bruker, 2008 ▶), *SAINT-Plus* (Bruker, 2008 ▶), *SHELXS97* and *SHELXL97* (Sheldrick, 2008 ▶), *ATOMS for Windows* (Dowty, 2006 ▶) and *publCIF* (Westrip, 2010 ▶).

## supplementary crystallographic information

### Crystal data

**Table:**

Pb_3_[IO_4_(OH)_2_]_2_	*F*(000) = 1808
*M**_r_* = 1071.40	*D*_x_ = 6.857 Mg m^−^^3^
Monoclinic, *P*2_1_/*c*	Mo *K*α radiation, λ = 0.71073 Å
Hall symbol: -P 2ybc	Cell parameters from 3673 reflections
*a* = 8.9653 (9) Å	θ = 3.2–30.5°
*b* = 9.2113 (9) Å	µ = 54.55 mm^−^^1^
*c* = 12.8052 (13) Å	*T* = 296 K
β = 101.042 (2)°	Block, colourless
*V* = 1037.90 (18) Å^3^	0.06 × 0.06 × 0.05 mm
*Z* = 4	

### Data collection

**Table:**

Siemens SMART CCD diffractometer	3196 independent reflections
Radiation source: fine-focus sealed tube	2587 reflections with *I* > 2σ(*I*)
Graphite monochromator	*R*_int_ = 0.000
ω scans	θ_max_ = 30.6°, θ_min_ = 2.3°
Absorption correction: multi-scan (*TWINABS*; Bruker, 2008)	*h* = −12→12
*T*_min_ = 0.253, *T*_max_ = 0.746	*k* = 0→13
3196 measured reflections	*l* = 0→18

### Refinement

**Table:**

Refinement on *F*^2^	Primary atom site location: structure-invariant direct methods
Least-squares matrix: full	Secondary atom site location: difference Fourier map
*R*[*F*^2^ > 2σ(*F*^2^)] = 0.041	H-atom parameters not refined
*wR*(*F*^2^) = 0.087	*w* = 1/[σ^2^(*F*_o_^2^) + (0.0319*P*)^2^ + 17.8096*P*] where *P* = (*F*_o_^2^ + 2*F*_c_^2^)/3
*S* = 1.07	(Δ/σ)_max_ < 0.001
3196 reflections	Δρ_max_ = 2.88 e Å^−^^3^
94 parameters	Δρ_min_ = −1.95 e Å^−^^3^
0 restraints	

### Special details

**Table:**

Geometry. All e.s.d.'s (except the e.s.d. in the dihedral angle between two l.s. planes) are estimated using the full covariance matrix. The cell e.s.d.'s are taken into account individually in the estimation of e.s.d.'s in distances, angles and torsion angles; correlations between e.s.d.'s in cell parameters are only used when they are defined by crystal symmetry. An approximate (isotropic) treatment of cell e.s.d.'s is used for estimating e.s.d.'s involving l.s. planes.
Refinement. Refinement of *F*^2^ against ALL reflections. The weighted *R*-factor *wR* and goodness of fit *S* are based on *F*^2^, conventional *R*-factors *R* are based on *F*, with *F* set to zero for negative *F*^2^. The threshold expression of *F*^2^ > σ(*F*^2^) is used only for calculating *R*-factors(gt) *etc*. and is not relevant to the choice of reflections for refinement. *R*-factors based on *F*^2^ are statistically about twice as large as those based on *F*, and *R*- factors based on ALL data will be even larger.

### Fractional atomic coordinates and isotropic or equivalent isotropic displacement parameters (Å^2^)

**Table:**

	*x*	*y*	*z*	*U*_iso_*/*U*_eq_	
Pb1	0.12192 (5)	0.13042 (4)	0.11904 (3)	0.01318 (10)	
Pb2	0.25685 (5)	0.51903 (5)	0.01540 (4)	0.01842 (11)	
Pb3	0.37507 (5)	0.36874 (5)	0.38134 (3)	0.01409 (10)	
I1	0.00670 (7)	0.23313 (7)	0.36046 (5)	0.00838 (13)	
I2	0.50186 (7)	0.24738 (6)	0.14267 (5)	0.00735 (13)	
O1	0.0343 (9)	0.3332 (8)	0.0015 (6)	0.0110 (14)*	
O2	0.0389 (9)	0.7922 (8)	0.2811 (6)	0.0125 (15)*	
O3	0.1058 (9)	0.4046 (8)	0.4090 (6)	0.0122 (15)*	
O4	0.1088 (10)	0.5549 (9)	0.1797 (6)	0.0178 (17)*	
O5	0.1831 (9)	0.8100 (8)	0.1159 (6)	0.0129 (15)*	
O6	0.1842 (9)	0.1429 (8)	0.3433 (6)	0.0117 (15)*	
O7	0.3161 (9)	0.3260 (8)	0.1618 (6)	0.0121 (15)*	
O8	0.4052 (9)	0.0832 (8)	0.0785 (6)	0.0121 (15)*	
O9	0.4802 (9)	0.3391 (8)	0.0121 (6)	0.0144 (16)*	
O10	0.5247 (9)	0.1474 (8)	0.2794 (6)	0.0145 (16)*	
O11	0.6146 (10)	0.3912 (8)	0.2170 (6)	0.0158 (16)*	
O12	0.6856 (10)	0.1562 (9)	0.1172 (6)	0.0182 (17)*	

### Atomic displacement parameters (Å^2^)

**Table:**

	*U*^11^	*U*^22^	*U*^33^	*U*^12^	*U*^13^	*U*^23^
Pb1	0.0134 (2)	0.01402 (19)	0.01215 (19)	−0.00236 (15)	0.00243 (15)	0.00042 (14)
Pb2	0.0168 (2)	0.01424 (19)	0.0236 (2)	0.00084 (17)	0.00251 (16)	0.00040 (16)
Pb3	0.0153 (2)	0.0173 (2)	0.01036 (18)	−0.00366 (16)	0.00426 (15)	−0.00050 (14)
I1	0.0075 (3)	0.0104 (3)	0.0072 (3)	−0.0003 (2)	0.0014 (2)	0.0006 (2)
I2	0.0066 (3)	0.0078 (3)	0.0076 (3)	−0.0002 (2)	0.0011 (2)	−0.0005 (2)

### Geometric parameters (Å, º)

**Table:**

Pb1—O1	2.433 (7)	Pb3—O9^vi^	2.599 (8)
Pb1—O7	2.493 (8)	Pb3—O6	2.678 (8)
Pb1—O2^i^	2.577 (8)	Pb3—O12^vii^	2.704 (8)
Pb1—O3^ii^	2.685 (7)	Pb3—O8^vii^	2.767 (8)
Pb1—O8	2.723 (8)	Pb3—O7	2.787 (7)
Pb1—O6	2.821 (7)	Pb3—O10	2.886 (8)
Pb1—O3^i^	2.888 (8)	I1—O6	1.845 (8)
Pb1—O5^iii^	3.004 (7)	I1—O3	1.860 (7)
Pb2—O7	2.564 (7)	I1—O2^i^	1.861 (7)
Pb2—O9	2.606 (8)	I1—O1^vi^	1.877 (7)
Pb2—O1	2.609 (7)	I1—O5^i^	1.920 (8)
Pb2—O6^ii^	2.638 (7)	I1—O4^i^	1.956 (8)
Pb2—O4	2.714 (8)	I2—O11	1.820 (8)
Pb2—O9^iv^	2.777 (8)	I2—O9	1.850 (8)
Pb2—O1^v^	2.915 (7)	I2—O8	1.855 (7)
Pb2—O5	3.099 (8)	I2—O7	1.874 (8)
Pb3—O8^vi^	2.527 (7)	I2—O12	1.932 (9)
Pb3—O3	2.528 (8)	I2—O10	1.954 (8)
			
O1—Pb1—O7	73.1 (2)	O7—I2—O10	90.5 (3)
O1—Pb1—O2^i^	73.6 (2)	O12—I2—O10	90.0 (3)
O7—Pb1—O2^i^	84.6 (2)	I1^ii^—O1—Pb1	108.2 (3)
O1—Pb1—O3^ii^	61.5 (2)	I1^ii^—O1—Pb2	103.7 (3)
O7—Pb1—O3^ii^	102.0 (2)	Pb1—O1—Pb2	108.0 (3)
O2^i^—Pb1—O3^ii^	129.7 (2)	I1^ii^—O1—Pb2^v^	97.4 (3)
O1—Pb1—O8	102.0 (2)	Pb1—O1—Pb2^v^	125.6 (3)
O7—Pb1—O8	61.3 (2)	Pb2—O1—Pb2^v^	111.1 (2)
O2^i^—Pb1—O8	144.9 (2)	I1^ii^—O1—Pb3^ii^	57.6 (2)
O3^ii^—Pb1—O8	70.3 (2)	Pb1—O1—Pb3^ii^	73.14 (18)
O1—Pb1—O6	125.2 (2)	Pb2—O1—Pb3^ii^	73.13 (17)
O7—Pb1—O6	75.7 (2)	Pb2^v^—O1—Pb3^ii^	154.3 (2)
O2^i^—Pb1—O6	59.4 (2)	I1^viii^—O2—Pb1^viii^	106.0 (3)
O3^ii^—Pb1—O6	170.7 (2)	I1^viii^—O2—Pb2^ix^	156.0 (4)
O8—Pb1—O6	101.0 (2)	Pb1^viii^—O2—Pb2^ix^	97.4 (2)
O1—Pb1—O3^i^	109.8 (2)	I1^viii^—O2—Pb1^x^	75.1 (2)
O7—Pb1—O3^i^	174.4 (2)	Pb1^viii^—O2—Pb1^x^	155.4 (3)
O2^i^—Pb1—O3^i^	91.7 (2)	Pb2^ix^—O2—Pb1^x^	86.03 (16)
O3^ii^—Pb1—O3^i^	83.5 (2)	I1^viii^—O2—Pb3^viii^	62.0 (2)
O8—Pb1—O3^i^	121.6 (2)	Pb1^viii^—O2—Pb3^viii^	78.83 (19)
O6—Pb1—O3^i^	98.8 (2)	Pb2^ix^—O2—Pb3^viii^	129.9 (2)
O1—Pb1—O5^iii^	141.7 (2)	Pb1^x^—O2—Pb3^viii^	80.39 (14)
O7—Pb1—O5^iii^	126.3 (2)	I1—O3—Pb3	104.5 (3)
O2^i^—Pb1—O5^iii^	134.1 (2)	I1—O3—Pb1^vi^	99.4 (3)
O3^ii^—Pb1—O5^iii^	81.0 (2)	Pb3—O3—Pb1^vi^	104.8 (3)
O8—Pb1—O5^iii^	70.2 (2)	I1—O3—Pb1^viii^	106.8 (3)
O6—Pb1—O5^iii^	93.0 (2)	Pb3—O3—Pb1^viii^	138.4 (3)
O3^i^—Pb1—O5^iii^	54.4 (2)	Pb1^vi^—O3—Pb1^viii^	96.5 (2)
O7—Pb2—O9	61.2 (2)	I1^viii^—O4—Pb2	102.2 (3)
O7—Pb2—O1	69.1 (2)	I1^viii^—O4—Pb3	146.5 (3)
O9—Pb2—O1	99.3 (2)	Pb2—O4—Pb3	98.2 (2)
O7—Pb2—O6^ii^	101.7 (2)	I1^viii^—O4—Pb1^viii^	71.6 (2)
O9—Pb2—O6^ii^	72.2 (2)	Pb2—O4—Pb1^viii^	173.4 (3)
O1—Pb2—O6^ii^	60.6 (2)	Pb3—O4—Pb1^viii^	88.39 (17)
O7—Pb2—O4	65.3 (2)	I1^viii^—O4—Pb2^v^	68.3 (2)
O9—Pb2—O4	125.5 (2)	Pb2—O4—Pb2^v^	87.5 (2)
O1—Pb2—O4	69.6 (2)	Pb3—O4—Pb2^v^	139.5 (2)
O6^ii^—Pb2—O4	129.6 (2)	Pb1^viii^—O4—Pb2^v^	87.94 (18)
O7—Pb2—O9^iv^	110.9 (2)	I1^viii^—O4—Pb1	143.6 (3)
O9—Pb2—O9^iv^	67.9 (3)	Pb2—O4—Pb1	72.12 (18)
O1—Pb2—O9^iv^	163.1 (2)	Pb3—O4—Pb1	68.44 (14)
O6^ii^—Pb2—O9^iv^	103.9 (2)	Pb1^viii^—O4—Pb1	111.3 (2)
O4—Pb2—O9^iv^	126.5 (2)	Pb2^v^—O4—Pb1	75.48 (15)
O7—Pb2—O1^v^	115.8 (2)	I1^viii^—O5—Pb1^x^	101.0 (3)
O9—Pb2—O1^v^	167.4 (2)	I1^viii^—O5—Pb2	90.7 (3)
O1—Pb2—O1^v^	68.9 (2)	Pb1^x^—O5—Pb2	155.4 (3)
O6^ii^—Pb2—O1^v^	97.3 (2)	I1^viii^—O5—Pb1^v^	69.1 (2)
O4—Pb2—O1^v^	55.7 (2)	Pb1^x^—O5—Pb1^v^	75.90 (16)
O9^iv^—Pb2—O1^v^	122.7 (2)	Pb2—O5—Pb1^v^	88.49 (18)
O7—Pb2—O5	109.1 (2)	I1^viii^—O5—Pb3^vii^	163.1 (3)
O9—Pb2—O5	141.5 (2)	Pb1^x^—O5—Pb3^vii^	92.88 (19)
O1—Pb2—O5	112.0 (2)	Pb2—O5—Pb3^vii^	80.25 (17)
O6^ii^—Pb2—O5	142.9 (2)	Pb1^v^—O5—Pb3^vii^	124.4 (2)
O4—Pb2—O5	53.0 (2)	I1—O6—Pb2^vi^	103.6 (3)
O9^iv^—Pb2—O5	84.2 (2)	I1—O6—Pb3	99.4 (3)
O1^v^—Pb2—O5	50.8 (2)	Pb2^vi^—O6—Pb3	103.9 (3)
O8^vi^—Pb3—O3	76.1 (3)	I1—O6—Pb1	97.6 (3)
O8^vi^—Pb3—O9^vi^	61.9 (2)	Pb2^vi^—O6—Pb1	142.8 (3)
O3—Pb3—O9^vi^	104.1 (2)	Pb3—O6—Pb1	102.2 (2)
O8^vi^—Pb3—O6	105.1 (2)	I2—O7—Pb1	107.0 (3)
O3—Pb3—O6	62.2 (2)	I2—O7—Pb2	103.7 (3)
O9^vi^—Pb3—O6	71.7 (2)	Pb1—O7—Pb2	107.6 (3)
O8^vi^—Pb3—O12^vii^	78.7 (2)	I2—O7—Pb3	100.6 (3)
O3—Pb3—O12^vii^	70.9 (3)	Pb1—O7—Pb3	108.2 (3)
O9^vi^—Pb3—O12^vii^	140.0 (2)	Pb2—O7—Pb3	127.7 (3)
O6—Pb3—O12^vii^	129.8 (2)	I2—O7—Pb3^ii^	56.96 (19)
O8^vi^—Pb3—O8^vii^	75.7 (3)	Pb1—O7—Pb3^ii^	72.30 (17)
O3—Pb3—O8^vii^	123.1 (2)	Pb2—O7—Pb3^ii^	73.10 (17)
O9^vi^—Pb3—O8^vii^	104.4 (2)	Pb3—O7—Pb3^ii^	154.9 (3)
O6—Pb3—O8^vii^	174.5 (2)	I2—O8—Pb3^ii^	104.5 (3)
O12^vii^—Pb3—O8^vii^	55.7 (2)	I2—O8—Pb1	99.1 (3)
O8^vi^—Pb3—O7	174.9 (2)	Pb3^ii^—O8—Pb1	103.7 (3)
O3—Pb3—O7	99.1 (2)	I2—O8—Pb3^xi^	104.1 (3)
O9^vi^—Pb3—O7	121.4 (2)	Pb3^ii^—O8—Pb3^xi^	104.3 (3)
O6—Pb3—O7	73.5 (2)	Pb1—O8—Pb3^xi^	137.3 (3)
O12^vii^—Pb3—O7	98.4 (2)	I2—O9—Pb3^ii^	102.0 (3)
O8^vii^—Pb3—O7	106.2 (2)	I2—O9—Pb2	102.9 (3)
O8^vi^—Pb3—O10	127.3 (2)	Pb3^ii^—O9—Pb2	107.1 (3)
O3—Pb3—O10	134.0 (2)	I2—O9—Pb2^iv^	112.6 (4)
O9^vi^—Pb3—O10	68.2 (2)	Pb3^ii^—O9—Pb2^iv^	118.5 (3)
O6—Pb3—O10	72.9 (2)	Pb2—O9—Pb2^iv^	112.1 (3)
O12^vii^—Pb3—O10	143.8 (2)	I2—O10—Pb3	95.4 (3)
O8^vii^—Pb3—O10	102.3 (2)	I2—O10—Pb2^xi^	148.8 (4)
O7—Pb3—O10	57.2 (2)	Pb3—O10—Pb2^xi^	98.9 (2)
O6—I1—O3	93.1 (3)	I2—O10—Pb3^xi^	79.3 (2)
O6—I1—O2^i^	92.8 (3)	Pb3—O10—Pb3^xi^	167.0 (3)
O3—I1—O2^i^	94.6 (3)	Pb2^xi^—O10—Pb3^xi^	91.50 (19)
O6—I1—O1^vi^	90.6 (3)	I2—O10—Pb1	67.0 (2)
O3—I1—O1^vi^	89.3 (3)	Pb3—O10—Pb1	78.32 (18)
O2^i^—I1—O1^vi^	174.7 (3)	Pb2^xi^—O10—Pb1	143.2 (2)
O6—I1—O5^i^	174.5 (3)	Pb3^xi^—O10—Pb1	88.65 (17)
O3—I1—O5^i^	90.9 (3)	I2—O11—Pb3	85.6 (3)
O2^i^—I1—O5^i^	90.5 (3)	I2—O11—Pb2^iv^	88.1 (3)
O1^vi^—I1—O5^i^	85.8 (3)	Pb3—O11—Pb2^iv^	157.4 (3)
O6—I1—O4^i^	90.9 (3)	I2—O11—Pb1^vii^	171.0 (4)
O3—I1—O4^i^	174.5 (3)	Pb3—O11—Pb1^vii^	95.8 (2)
O2^i^—I1—O4^i^	89.0 (3)	Pb2^iv^—O11—Pb1^vii^	93.74 (19)
O1^vi^—I1—O4^i^	86.9 (3)	I2—O11—Pb2	64.5 (2)
O5^i^—I1—O4^i^	84.8 (3)	Pb3—O11—Pb2	83.53 (18)
O11—I2—O9	95.3 (3)	Pb2^iv^—O11—Pb2	74.20 (15)
O11—I2—O8	172.0 (3)	Pb1^vii^—O11—Pb2	124.5 (2)
O9—I2—O8	90.7 (3)	I2—O12—Pb3^xi^	104.2 (4)
O11—I2—O7	93.9 (4)	I2—O12—Pb2^iv^	85.5 (3)
O9—I2—O7	90.0 (3)	Pb3^xi^—O12—Pb2^iv^	151.9 (3)
O8—I2—O7	91.2 (3)	I2—O12—Pb3^ii^	68.4 (2)
O11—I2—O12	89.9 (4)	Pb3^xi^—O12—Pb3^ii^	79.9 (2)
O9—I2—O12	89.5 (4)	Pb2^iv^—O12—Pb3^ii^	79.42 (16)
O8—I2—O12	84.9 (3)	I2—O12—Pb1^xii^	155.4 (4)
O7—I2—O12	176.1 (3)	Pb3^xi^—O12—Pb1^xii^	98.2 (2)
O11—I2—O10	85.5 (3)	Pb2^iv^—O12—Pb1^xii^	79.41 (17)
O9—I2—O10	179.1 (3)	Pb3^ii^—O12—Pb1^xii^	126.6 (2)
O8—I2—O10	88.4 (3)		

Symmetry codes: (i) −*x*, *y*−1/2, −*z*+1/2; (ii) *x*, −*y*+1/2, *z*−1/2; (iii) *x*, *y*−1, *z*; (iv) −*x*+1, −*y*+1, −*z*; (v) −*x*, −*y*+1, −*z*; (vi) *x*, −*y*+1/2, *z*+1/2; (vii) −*x*+1, *y*+1/2, −*z*+1/2; (viii) −*x*, *y*+1/2, −*z*+1/2; (ix) *x*, −*y*+3/2, *z*+1/2; (x) *x*, *y*+1, *z*; (xi) −*x*+1, *y*−1/2, −*z*+1/2; (xii) *x*+1, *y*, *z*.

### Hydrogen-bond geometry (Å)

**Table:**

*D*—H···*A*	*D*···*A*
O4···O7	2.849 (11)
O4···O2^i^	2.849 (11)
O5···O11^vii^	2.634 (11)
O10···O11^xi^	2.675 (11)
O12···O2^xi^	2.852 (11)

Symmetry codes: (i) −*x*, *y*−1/2, −*z*+1/2; (vii) −*x*+1, *y*+1/2, −*z*+1/2; (xi) −*x*+1, *y*−1/2, −*z*+1/2.
